# Patient eligibility for trials with imaging response assessment at the time of molecular tumor board presentation

**DOI:** 10.1186/s40644-024-00708-5

**Published:** 2024-06-07

**Authors:** Nabeel Mansour, Kathrin Heinrich, Danmei Zhang, Michael Winkelmann, Maria Ingenerf, Lukas Gold, Konstantin Klambauer, Martina Rudelius, Frederick Klauschen, Michael von Bergwelt-Baildon, Jens Ricke, Volker Heinemann, C. Benedikt Westphalen, Wolfgang G. Kunz

**Affiliations:** 1 Department of Radiology, University Hospital, LMU Munich, Marchioninistr. 15, 81377 Munich, Germany; 2 Department of Medicine III, University Hospital, LMU Munich, Munich, Germany; 3 Comprehensive Cancer Center München-LMU (CCCM LMU), LMU Munich, Munich, Germany; 4 Institute of Pathology, Ludwig-Maximilians-Universität Munich, Munich, Germany; 5 German Cancer Consortium (DKTK partner site Munich), Heidelberg, Germany

**Keywords:** RECIST, Response assessment, MTB, Precision oncology

## Abstract

**Purpose:**

To assess the eligibility of patients with advanced or recurrent solid malignancies presented to a molecular tumor board (MTB) at a large precision oncology center for inclusion in trials with the endpoints objective response rate (ORR) or duration of response (DOR) based on Response Evaluation Criteria in Solid Tumors (RECIST version 1.1).

**Methods:**

Prospective patients with available imaging at the time of presentation in the MTB were included. Imaging data was reviewed for objectifiable measurable disease (MD) according to RECIST v1.1. Additionally, we evaluated the patients with MD for representativeness of the identified measurable lesion(s) in relation to the overall tumor burden.

**Results:**

262 patients with different solid malignancies were included. 177 patients (68%) had MD and 85 (32%) had non-measurable disease (NMD) at the time point of MTB presentation in accordance with RECIST v1.1. MD was not representative of the overall tumor burden in eleven patients (6%). The main reasons for NMD were lesions with longest diameter shorter than 10 mm (22%) and non-measurable peritoneal carcinomatosis (18%). Colorectal cancer and malignant melanoma displayed the highest rates of MD (> 75%). In contrast, gastric cancer, head and neck malignancies, and ovarian carcinoma had the lowest rates of MD (< 55%). In case of MD, the measurable lesions were representative of the overall tumor burden in the vast majority of cases (94%).

**Conclusion:**

Approximately one third of cancer patients with advanced solid malignancies are not eligible for treatment response assessment in trials with endpoints ORR or DOR at the time of MTB presentation. The rate of patients eligible for trials with imaging endpoints differs significantly based on the underlying malignancy and should be taken under consideration during the planning of new precision oncology trials.

## Background

Next generation sequencing has (NGS) enabled the identification of molecularly guided treatment options for patients with cancer [1]. In comprehensive cancer centers, patients with advanced cancer and upon progression on systemic therapy are increasingly presented in a molecular tumor board (MTB) after NGS for possible inclusion in clinical trials [2]. With targeted therapies on the rise, MTBs serve as a central platform for allocating personalized treatments [3]. For the assessment of the safety and efficacy of such treatments, clinical trials are critical. The evidence for efficacy of targeted treatment is often based on non-randomized trials with the endpoints such as objective response rate (ORR), disease-free survival (DFS) and progression-free survival (PFS) [4–7]. Patient inclusion in trials is therefore very often dependent on objectifiable tumor burden in oncologic imaging. MTBs can and should serve as a platform to identify patients for early clinical trials and trials investigating targeted treatments [8].

The importance of objective tumor response assessment led to the development of systems used to standardize the determination and communication of the impact of a treatment on tumor burden. In the context of evaluating solid tumor response or progression in clinical trials, the prevailing standard are the Response Evaluation Criteria in Solid Tumors (RECIST) [9]. RECIST was developed with the objective of simplifying measurement of tumor burden and to limit the potential for overestimation of response rates [9]. In 2009, revisions were made (RECIST 1.1) incorporating major changes [9] followed by an updated version with clarifications published in 2016 from the RECIST committee [10]. These guidelines require serial imaging with protocol-specified frequency and imaging modality [11].

The utilization of tumor regression as the primary endpoint in phase II trials, aimed at assessing novel agents for indications of anti-tumor efficacy, is substantiated by an extensive body of evidence over several years. This evidence implies that, for numerous solid tumors, agents capable of inducing tumor shrinkage in a subset of patients exhibit a reasonable, albeit not flawless, likelihood of subsequently revealing enhancements in overall survival or other time-to-event metrics in randomized phase III studies [12–14] with the caveat that the surrogacy of ORR and PFS for overall survival (OS) differs based on treatment and tumor [15, 16]. Moreover, to advance drug development, clinical trials conducted in advanced disease contexts are progressively incorporating time to progression (TTP) or PFS as an endpoint for deriving efficacy assessments at both the phase II and phase III stages [17–19]. This approach is also founded on anatomical measurements of tumor size.

The oncology community should be cognizant of the fact that trials with imaging-based endpoints only relate to patients with measurable disease. Many targeted therapy studies such as the KEYNOTE-158 study relied on RECIST assessment for the inclusion of eligible patients with measurable disease [20, 21]. So far, the proportion of RECIST-eligible patients in MTB is unknown, and there is no literature on the influence of tumor entity or metastatic phenotype on the inclusion rate in trials with targeted therapies. With targeted treatment trials on the rise, we aimed to assess the eligibly of patients with solid malignancies who presented to a large precision oncology center based on RECIST version 1.1, and the influence of tumor-specific metastatic phenotypes.

## Methods

### Study design and population

All patients with solid malignancies included in this retrospective single-center study were presented in the molecular tumor board at the Comprehensive Cancer Center München-LMU (CCCM^LMU^). In 2019, in-house diagnostics were changed to a 161-gene panel (Oncomine™ Comprehensive Assay v3 (OCAv3), ThermoFisher Scientific) and the Oncomine Tumor Mutational Load Assay (ThermoFisher Scientific) was added to the diagnostic repertoire [22]. For the present analysis, we only included patients that received the 161-gene panel and Tumor Mutational Analysis. Inclusion criteria was current cross-sectional imaging within the clinical routine no longer than three months prior to case presentation in the MTB. The study was conducted in accordance with the principles of the Declaration of Helsinki and International Council for Harmonisation Good Clinical Practice guidelines. All patients gave written informed consent, and the study protocol was approved by the Ethics Committee of the medical faculty of the Ludwig Maximilians University Munich. Furthermore, all molecular diagnostic tests were conducted in accordance with the medical treatment contract signed by each patient.

### Evaluation of tumor burden

Overall tumor burden assessment was performed in a sequential manner by two radiologists with extensive experience in cross sectional oncological imaging based on RECIST version 1.1 [23]. Computed tomography (CT) and magnetic resonance imaging (MRI) were evaluated for the presence or absence of measurable disease (MD) in the scan with closest to the time point prior of case presentation at the MTB. Furthermore, in cases with simultaneous MD and non-measurable disease (NMD), the MD cohort was also assessed in regards of representativeness of overall tumor burden.

### Definition of measurable disease

**Fig. 1:**
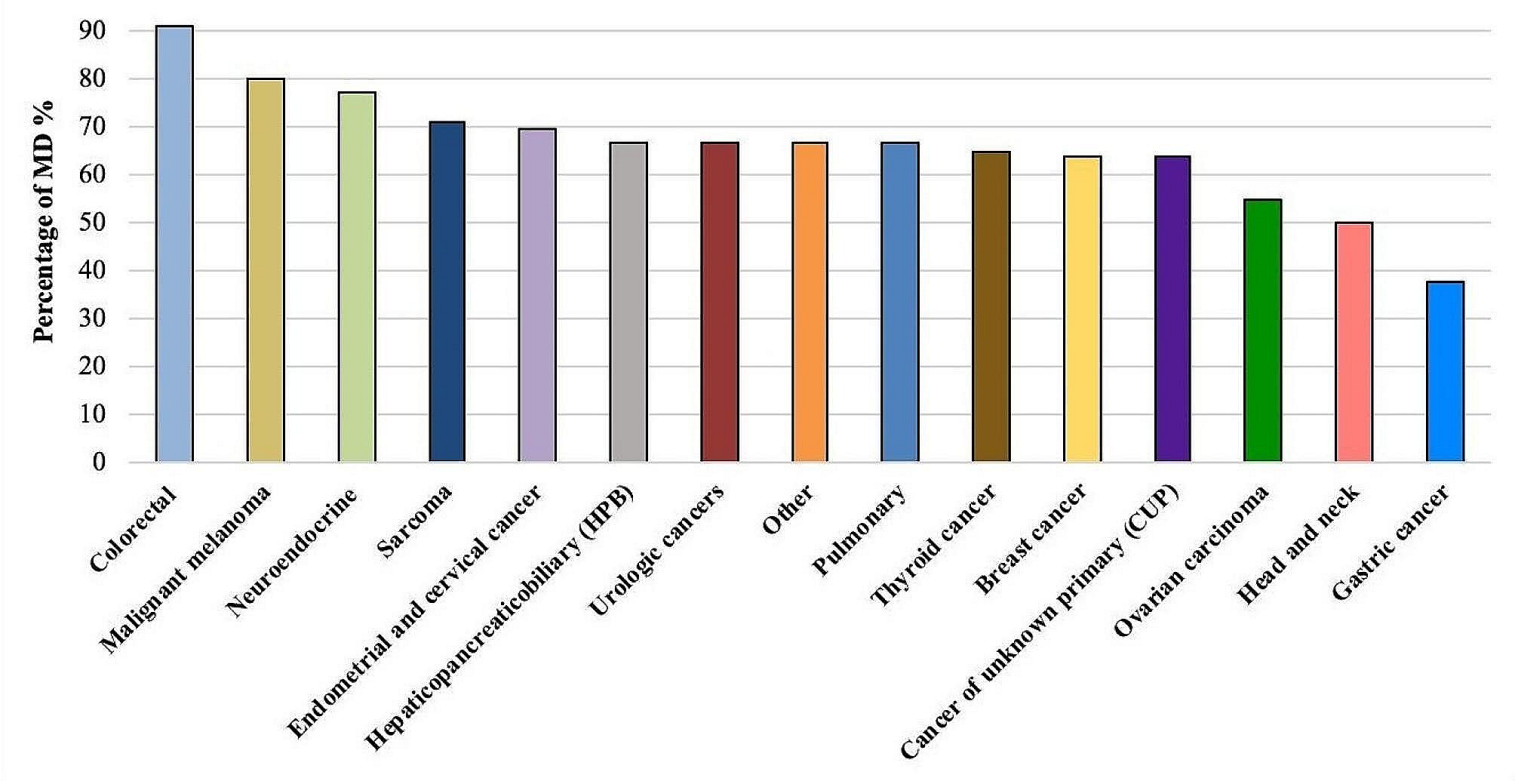
Distribution of measurable based on the underlying tumor entity with highest rate of MD from left to right. MD = measurable disease; NMD = non-measurable disease

MD is defined by the presence of at least one measurable lesion. Measurable lesions were accurately measured in at least one dimension (longest diameter in the plane of measurement is to be recorded) with a minimum size of 10 mm by CT scan (slice thickness no greater than 5 mm). Malignant lymph nodes were classified as measurable when pathologically enlarges with ≥ 15 mm in short axis when assessed by CT scan (slice thickness no greater than 5 mm). The distribution of MD based on the underlying solid tumor entity is shown in Fig. 1.

### Definition of non-measurable disease

All other lesions, including small lesions which did not fit the above-mentioned criteria and truly non-measurable lesion like leptomeningeal disease, ascites, pleural or pericardial effusion etc. were categorized as non-measurable. Tumor lesions subjected to local treatment or in an area subjected to other loco-regional therapy were usually not considered measurable unless there has been demonstrated progression in the lesion. Blastic bone lesions were also considered non-measurable.

### Evaluation of representativeness of MD in regards to the overall tumor burden

**Fig. 2:**
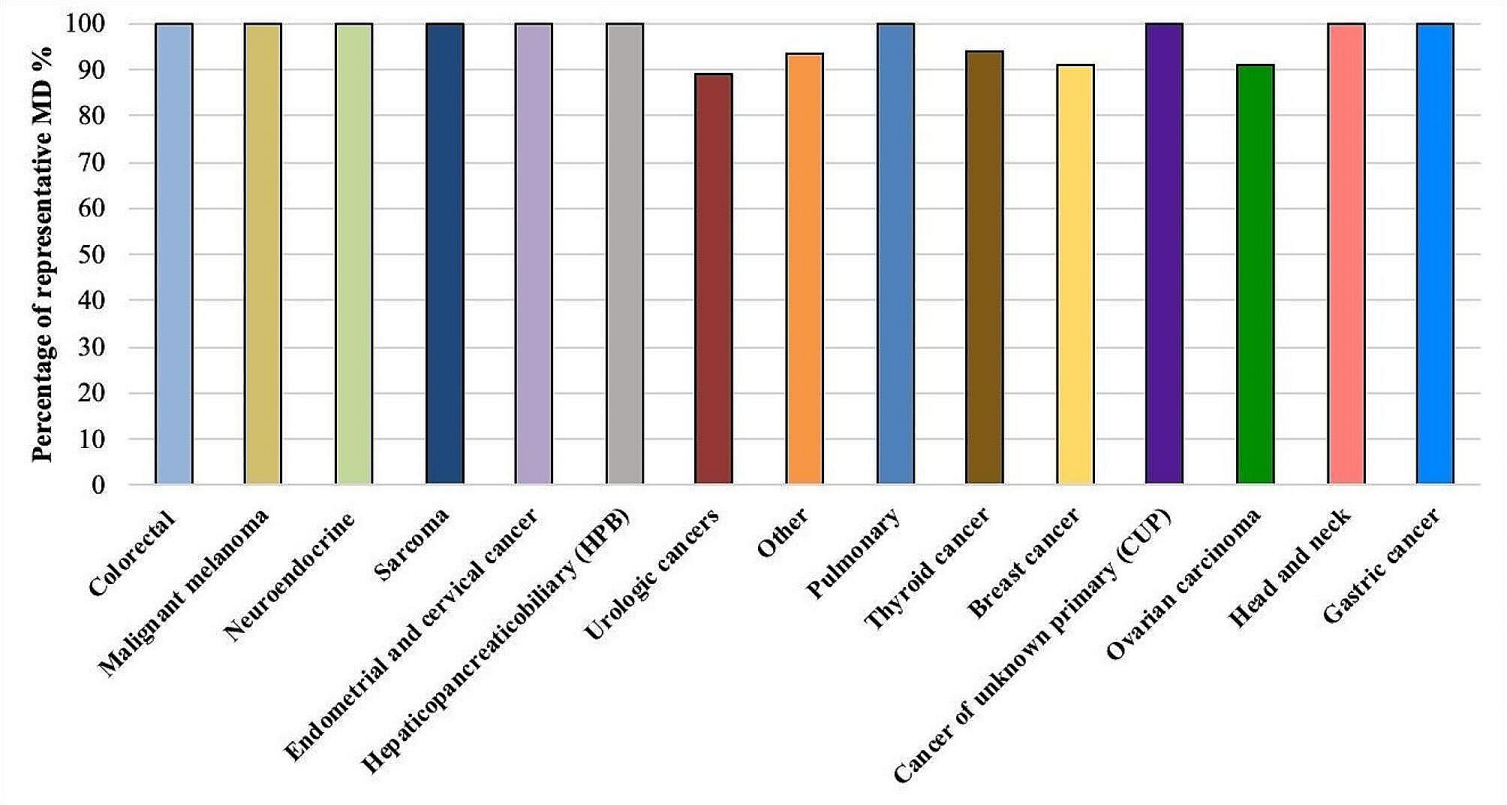
Rate of MD representative of the overall tumor burden across the included solid tumor entities. In most cases, measurable disease (MD) accurately represents the overall tumor burden; however, in 6% of cases, it is not representative due to concomitant extensive non-measurable disease (NMD), as demonstrated in a thyroid cancer case in Fig. 3. MD = measurable disease; NMD = non-measurable disease

Patients with limited measurable lesion(s) and simultaneous presence of unequivocal extensive NMD such as advanced peritoneal carcinomatosis or disseminated osteoblastic metastases were classified as MD non-representative of the overall tumor burden. An exemplary case of MD which was not representative of the overall tumor burden at the time of MTB is displayed in Fig. 2. In this case of resected thyroid cancer with a solitary measurable cervical nodal metastasis on the left side, extensive small nodular lung metastasis which are non-measurable due to small lesion size less than 10 mm are observed.

**Fig. 3:**
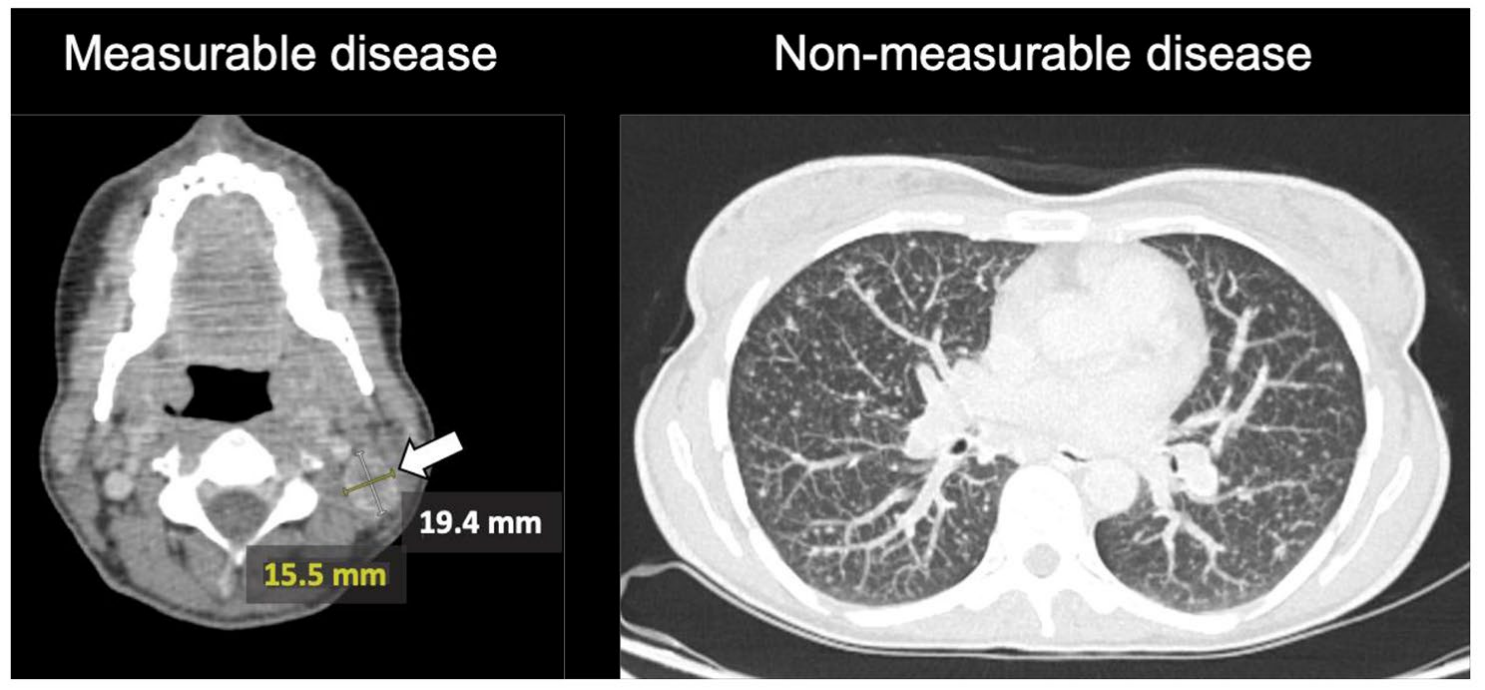
Example of MD non-representative of the overall tumor burden in contrast enhanced CT A female patient with resected thyroid cancer and a solitary measurable cervical lymph node metastasis on the left side (Level IIb) measuring 15,5 mm in the short axis (left image, white arrow). The CT-scan of the thorax in lung window (right image) of the same patient shows presence of extensive small nodular pulmonary metastases classified as non-measurable due to small tumor size of less than 10 mm

## Results

### Patient population

Imaging data of 302 patients with solid malignancies presented at the molecular tumor board (MTB) at the Comprehensive Cancer Center München-LMU (CCCM^LMU^) in the years 2019 to 2021 was reviewed. 262 patients with a median age of 55 years (19–83) and imaging less than three months prior to case presentation to the MTB (Median of 48 days) were included (Table 1). 177 (68%) patients had MD and 85 NMD (32%). No significant differences in age and sex were observed between the MD and NMD cohorts. As seen in Figs. 1 and 2, the solid tumor entities were summarized in 15 categories. The most common solid tumor entity included was breast cancer (*n* = 55, 20%). Analysis of previous therapy prior to MTB revealed that the patients in MD-cohort had significantly higher median of lines of systemic therapy compared to NMD-cohort (*p* = 0.005). In contrary, the NMD-cohort had a significantly higher median of surgical tumor resection prior to case presentation (*p* = 0.015). 24 (9%) of the patients were included in clinical trials based on the recommendation of MTB.

**Table 1:** Patient characteristics

	MD (*n* = 177)^1^	NMD (*n* = 85)^1^	*p*-value^2^
Age	56 (48, 64)	55 (47, 63)	0.6
Sex			0.13
Female	110 (62%)	61 (72%)	
Male	67 (38%)	24 (28%)	
Previous therapy before MTB			
Lines of systemic therapy	2.00 (1.00, 4.00)	2.00 (1.00, 3.00)	0.005*
Surgery	123 (69%)	71 (84%)	0.015*
Radiation therapy	77 (44%)	41 (48%)	0.5
First diagnosis to MTB (months)	28 (10, 55)	28 (10, 50)	0.8
Last imaging to MTB (days)	48 (33, 75)	47 (27, 80)	0.4
Included tumor entities			
Breast cancer	35 (64%)	20 (36%)	
Colorectal	29 (91%)	3 (9%)	
Hepatopancreaticobiliary	18 (67%)	9 (33%)	
Ovarian carcinoma	12 (55%)	10 (45%)	
Urologic cancers	12 (67%)	6 (33%)	
Sarcoma	12 (71%)	5 (29%)	
Thyroid cancer	11 (65%)	6 (35%)	
Other	10 (67%)	5 (33%)	
Neuroendocrine	10 (77%)	3 (23%)	
Endometrial and cervical cancer	9 (69%)	4 (31%)	
Cancer of unknown primary	7 (64%)	4 (36%)	
Gastric cancer	3 (38%)	5 (62%)	
Head and neck	3 (50%)	3 (50%)	
Malignant melanoma	4 (80%)	1 (20%)	
Pulmonary	2 (67%)	1 (33%)	

NMD, non-measurable disease; MD, measurable disease; MTB, molecular tumor board

^*1*^ Median (IQR); n (%)

^*2*^ Wilcoxon rank sum test; Pearson’s Chi-squared test

* Indicates a *p*-value lower than 0.05

### Measurable disease

Most of the included solid tumor entities (10 out of 15) displayed MD within the range of 50–75% (Fig. 1). Colorectal cancer (*n* = 32), malignant melanoma (*n* = 5) and neuroendocrine tumors (*n* = 13) had the highest rates of MD (91%, 80% and 77%, respectively). Solid tumor entities with the lowest rate of MD included were ovarian carcinoma, head and neck tumors and gastric cancer (< 55% MD). Information regarding the number of RECIST available lesions, mean lesion size and location are summarized per tumor entity in Table 2.

**Table 2:** Characteristics of measurable lesions

Tumor entity	MD (*n* = 177)^1^	Patients with ≥ 10 RECIST available lesions ^1^	Patients with 1–9 RECIST available lesions ^1^	Number of lesions $\varnothing$^2^	Lesion size $\varnothing$ (mm) ^2^	Number of affected sites $\varnothing$^†2^
Breast cancer	35 (64%)	12 (34%)	23 (66%)	3 (2.4)	21 (9.5)	2 (0.5)
Colorectal	29 (91%)	15 (52%)	14 (48%)	4 (2.6)	28 (12.9)	2 (0.7)
Hepatopancreaticobiliary	18 (67%)	9 (50%)	9 (50%)	2 (1.3)	28 (15.5)	2 (1.1)
Ovarian carcinoma	12 (55%)	3 (25%)	9 (75%)	4 (2.3)	34 (17.6)	2 (0.6)
Urologic cancers	12 (67%)	3 (25%)	9 (75%)	3 (1.5)	27 (12)	2 (0.9)
Sarcoma	12 (71%)	5 (42%)	7 (58%)	3 (2.2)	47 (19.5)	3 (1.3)
Thyroid cancer	11 (65%)	3 (27%)	8 (73%)	4 (2.1)	22 (5.7)	3 (1.1)
Other	10 (67%)	1 (10%)	9 (90%)	3 (2.0)	31 (20.7)	2 (0.9)
Neuroendocrine	10 (77%)	7 (70%)	3 (30%)	1 (0.5)	33 (2.9)	3 (0.8)
Endometrial and cervical cancer	9 (69%)	1 (11%)	8 (89%)	4 (2.7)	29 (17.2)	2 (0.8)
Cancer of unknown primary	7 (64%)	3 (43%)	4 (57%)	6 (2.7)	24 (5.0)	2 (1.3)
Gastric cancer	3 (38%)	/	3 (100%)	3 (0.8)	17 (2.2)	1 (0.5)
Head and neck	3 (50%)	/	3 (100%)	2 (0.5)	23 (6.6)	2 (0.9)
Malignant melanoma	4 (80%)	1 (25%)	3 (75%)	4 (0.9)	39 (22.5)	3 (0.5)
Pulmonary	2 (67%)	1 (50%)	1 (50%)	7 (n.a)	23 (n.a)	2 (n.a)

MD, measurable disease; NMD, non-measurable disease; SD, standard deviation

^*1*^ n (%); ^2^ n (SD); Mean number of lesions and mean lesion size were calculated in patients with 1–9 RECIST available lesion; ^†^ The number of affected sites was documented for MD and NMD and was listed as followed; liver, lung, lymphatic system, bone, local recurrence (non-liver or pulmonary), or other (e.g. peritoneal)

### Non-measurable disease

The most common cause of NMD was a lesion size less than 10 mm (22%). Non-measurable peritoneal carcinomatosis (18%) and post-therapeutic changes to target lesions resulting in non-measurability (15%) were the second and third most common reason, respectively. Some cancer entities presented with more frequent metastatic pattern of non-measurability. This was the case for non-measurable peritoneal carcinomatosis commonly observed in cases of advanced ovarian cancer (*n* = 6/22, 27%). Osteoblastic metastases were also a common reason for NMD (14% overall) with most cases observed in patients with breast cancer (*n* = 13/55, 24%). Eight of all patients with breast cancer (15%) had only osteoblastic metastases and therefore were classified as NMD.

### Measurable disease non-representative of tumor burden

**Fig. 4:**
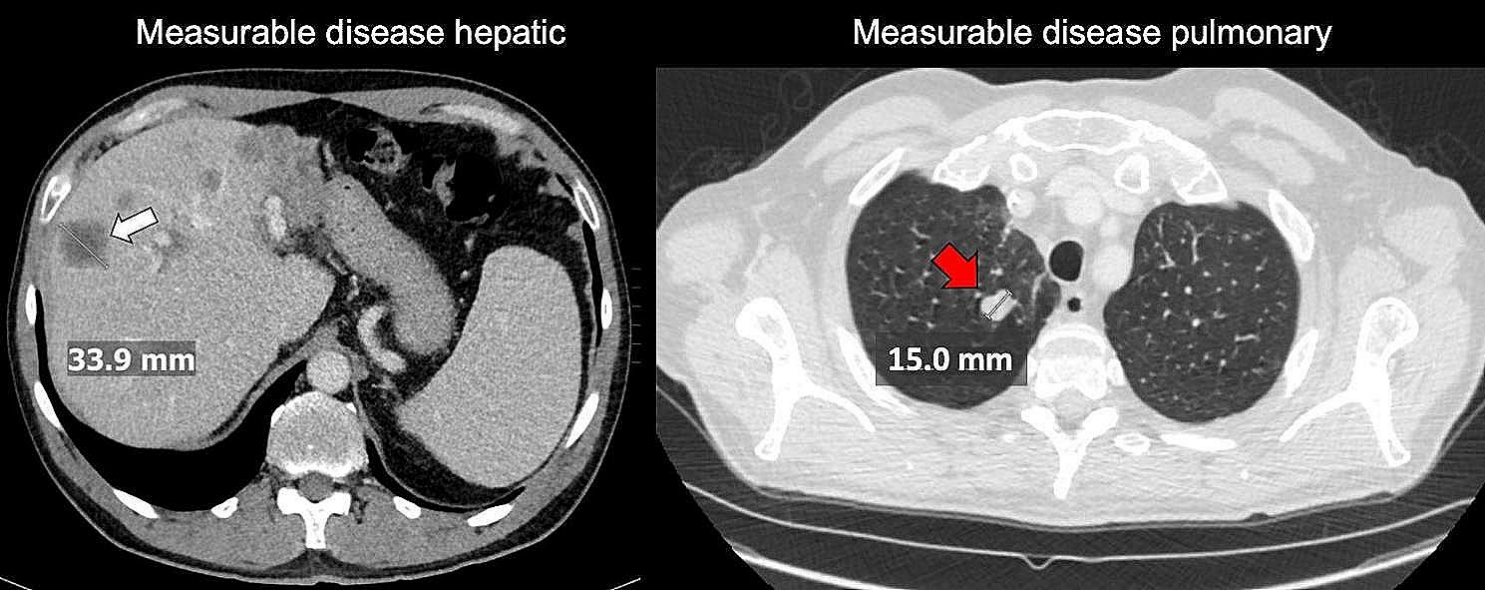
Exemplary cases of MD representative of the overall tumor burden in contrast enhanced CT Left image: Patient with recurrent colorectal cancer after surgery with multiple metachronous liver metastases. An axial CT-image of the liver in soft tissue window displays a well-defined liver metastasis in liver segment VIII is measured (white arrow). Right image: Axial CT image in pulmonary window in a patient with resected sarcoma and well-defined measurable pulmonary metastases. A well-defined measurable lesion in the right upper pulmonary lobe is displayed (red arrow, 15 mm)

Eleven patients (6%) had MD that was not representative of the overall tumor burden (Fig. 2). The patients were categorized in this group in the case of solitary measurable target lesions according to RECIST and simultaneous presence of extensive NMD such as extensive non-measurable peritoneal carcinomatosis or disseminated small metastases (pulmonary or hepatic). The majority of these patients presented either with prostate or breast cancer with solitary measurable target lesions and extensive non-measurable osteoblastic metastases (*n* = 8). Two patients had progressive ovarian cancer with solitary liver metastases as MD and extensive peritoneal carcinomatosis as NMD. Patient examples are provided in Figs. 4 and 3.

## Discussion

Although presenting with measurable disease at baseline, pretreated patients with disease progression, may not always be eligible for inclusion in clinical trials with targeted therapies due to NMD. The aim of this analysis was to evaluate patients with different solid malignancies from a large precision oncology center MTB regarding their eligibility for inclusion in clinical trials based on RECIST v1.1.

We discovered that approximately one third of cancer patients with advanced solid malignancies are not eligible for treatment response assessment in trials with the endpoints ORR or DOR due to NMD at the time of MTB presentation. Furthermore, we observed a high variability in the rate of eligibility at the time of case presentation based on the underlying solid malignancy as certain tumor-specific patterns were observed in several tumor entities affecting the assessment.

Specifically, several solid tumor entities like colorectal cancer presented with a high rate of MD (> 90%). This can be explained by the fact that high stage, recurrent or progressive colorectal cancer often affects the liver, thus presenting with well measurable liver metastases (69% of all colorectal cancer patients included in this study). In contrast, gastric cancer, head and neck tumors and ovarian cancer displayed the lowest rates of MD (< 55%). One explanation is the high rate of non-measurable peritoneal carcinomatosis in advanced ovarian or gastric cancer [24, 25]. Analysis of previous lines of therapy revealed a significantly higher rate of surgical treatment prior to MTB in the NMD-cohort. This can pose as a possible explanation for NMD, as post-surgical changes or even complete resection result in the tumor no longer being measurable. We also discovered that a small percentage of the MD cohort had measurable lesion(s) that were however not representative of the overall tumor burden (6%). This was determined for cases with isolated MD and predominant NMD.

Endpoints which rely on anatomical measurements such as ORR and DOR in patients with solid malignancies are important in the assessment of the tumor burden after treatment as they often serve as a primary or secondary end-point in clinical trials in order to generate evidence regarding efficacy [5, 26]. It has been shown that subjective assessment of tumor response may overestimate benefit and limit the potential role of real-world evidence [11, 27]. This highlights the importance of standardized objective response criteria such as RECIST. Although imperfect, RECIST carry a body of evidence greater than any other biomarker supporting its utility.

Multidisciplinary tumor boards (MDT) consist of a team of experts which are required to manage a patient from diagnosis to treatment and to discuss patients’ eligibility for clinical trials [28–37]. A recent study exploring the impact of MDTs for the inclusion of patients in two large comprehensive cancer centers in Munich (CCCM) has shown that MDTs result in increased inclusion of patients in oncological clinical trials [38]. The core composition of MDTs may vary depending on the cancer type, but it generally includes clinical oncologists, surgeons, pathologists, palliative care physicians, radiation oncologists and diagnostic and interventional radiologists [29]. A systematic review of MTBs in clinical practice has reviewed multiple studies of global MTBs. It has shown that radiologists were only present in the MTB in five of the 25 studies (20%) [3].

Of note, all patients in this study had progressive advanced-stage cancer with a low inclusion rate in clinical trials (9%). This aligns with data from other cancer centers, emphasizing the need for closer collaboration with early clinical trial programs to maximize benefits for patients undergoing comprehensive genomic profiling [22]. The low inclusion rate in trials can be attributed to various reasons. As reported, 32% of the patients lacked measurable target lesions during the Molecular Tumor Board (MTB) evaluation. From a clinical standpoint, the primary reason for disqualifying patients from clinical trials was the absence of druggable mutations or insufficient evidence supporting certain therapies. Additionally, patients often did not receive experimental treatment due to rapid clinical deterioration and advanced disease progression in end-stage cancer.

This study underscores the pivotal role of imaging in RECIST-eligibility assessment, given that most therapy trials require measurable lesions as an inclusion criterion. For tumor entities with a higher rate of non-measurable disease, new serological, pathological or imaging biomarkers are essential. The oncologic community should be cognizant of the significant variability in RECIST-eligibility based on tumor entity and metastatic phenotype, posing a potential limiting factor for trial inclusion.

### Limitations

The data is limited to a single-center with limited sample size, hence the representation of tumor entities may differ in larger cohorts.

## Conclusion

A substantial proportion of patients with refractory or progressive solid malignancies do not qualify for treatment trials with the endpoints ORR, DFS or PFS at the time of case presentation in MTB due to NMD. The underlying malignancy and tumor-specific metastatic phenotype affect the rate of RECIST-eligibility with a high level of variance. If MD is present, there is a high rate of it being representative of the present total tumor burden. These findings should be taken into consideration during the planning of new precision oncology trials.

## Data Availability

The datasets generated during and/or analysed during the current study are available from the corresponding author on reasonable request.

## Abbreviations

CCCM^LMU^: Comprehensive Cancer Center München-LMU
CT: Computed tomography
DOR: Duration of response
MD: Measurable disease
MDT: Multidisciplinary tumor board
MRI: Magnetic resonance imaging
MTB: Molecular tumor board
NGS: Next generation sequencing
NMD: Non-measurable disease
ORR: Objective response rate
PFS: Progression-free survival
RECIST: Response Evaluation Criteria in Solid Tumors
TTP: Time to progression

## References

[1] Prasad V, Fojo T, Brada M (2016). Precision oncology: origins, optimism, and potential. Lancet Oncol.

[2] Hoadley KA, Yau C, Wolf DM, Cherniack AD, Tamborero D, Ng S (2014). Multiplatform analysis of 12 cancer types reveals molecular classification within and across tissues of origin. Cell.

[3] Luchini C, Lawlor RT, Milella M, Scarpa A (2020). Molecular tumor boards in clinical practice. Trends Cancer.

[4] Wang C-Y, Wei L-Q, Niu H-Z, Gao W-Q, Wang T, Chen S-J (2018). Agitation thrombolysis combined with catheter-directed thrombolysis for the treatment of non-cirrhotic acute portal vein thrombosis. World J Gastroenterol.

[5] Aykan NF, Özatlı T (2020). Objective response rate assessment in oncology: current situation and future expectations. World J Clin Oncol.

[6] Lebwohl D, Kay A, Berg W, Baladi JF, Zheng J (2009). Progression-free survival: gaining on overall survival as a gold Standard and AcceleratingDrug Development. Cancer J.

[7] Delgado A, Guddati AK (2021). Clinical endpoints in oncology-a primer. Am J cancer Res.

[8] Dienstmann R, Garralda E, Aguilar S, Sala G, Viaplana C, Ruiz-Pace F et al. Evolving Landscape of molecular prescreening strategies for oncology early clinical trials. JCO Precis Oncol. 2020;4. 10.1200/PO.19.00398 PMC7446427 32923891

[9] Eisenhauer EA, Therasse P, Bogaerts J, Schwartz LH, Sargent D, Ford R (2009). New response evaluation criteria in solid tumours: revised RECIST guideline (version 1.1). Eur J Cancer.

[10] Schwartz LH, Litière S, De Vries E, Ford R, Gwyther S, Mandrekar S (2016). RECIST 1.1—Update and clarification: from the RECIST committee. Eur J Cancer.

[11] Feinberg BA, Zettler ME, Klink AJ, Lee CH, Gajra A, Kish JK (2021). Comparison of solid tumor treatment response observed in clinical practice with response reported in clinical trials. JAMA Netw Open.

[12] Paesmans M, Sculier J, Libert P, Bureau G, Dabouis G, Thiriaux J (1997). Response to chemotherapy has predictive value for further survival of patients with advanced non-small cell lung cancer: 10 years experience of the European Lung Cancer Working Party. Eur J Cancer.

[13] Goffin J, Baral S, Tu D, Nomikos D, Seymour L (2005). Objective responses in patients with malignant melanoma or renal cell cancer in early clinical studies do not predict regulatory approval. Clin Cancer Res.

[14] El-Maraghi RH, Eisenhauer EA (2008). Review of phase II trial designs used in studies of molecular targeted agents: outcomes and predictors of success in phase III. J Clin Oncol.

[15] Walia A, Haslam A, Prasad V (2022). FDA validation of surrogate endpoints in oncology: 2005–2022. J Cancer Policy.

[16] Shahnam A, Hitchen N, Nindra U, Manoharan S, Desai J, Tran B (2024). Objective response rate and progression-free survival as surrogates for overall survival treatment effect: a meta-analysis across diverse tumour groups and contemporary therapies. Eur J Cancer.

[17] Li L, Pan Z (2018). Progression-free survival and time to progression as real surrogate end points for overall survival in advanced breast cancer: a meta-analysis of 37 trials. Clin Breast Cancer.

[18] Hotta K, Fujiwara Y, Matsuo K, Kiura K, Takigawa N, Tabata M (2009). Time to progression as a surrogate marker for overall survival in patients with advanced non-small cell lung cancer. J Thorac Oncol.

[19] Burzykowski T, Buyse M, Piccart-Gebhart MJ, Sledge G, Carmichael J, Lück H-J et al. Evaluation of tumor response, disease control, progression-free survival, and time to progression as potential surrogate end points in metastatic breast cancer. 2008. 10.1200/JCO.2007.10.8407 18421050

[20] Jänne PA, Riely GJ, Gadgeel SM, Heist RS, Ou S-HI, Pacheco JM (2022). Adagrasib in non–small-cell lung cancer harboring a KRASG12C mutation. N Engl J Med.

[21] Marabelle A, Le DT, Ascierto PA, Di Giacomo AM, De Jesus-Acosta A, Delord J-P (2020). Efficacy of pembrolizumab in patients with noncolorectal high microsatellite instability/mismatch repair–deficient cancer: results from the phase II KEYNOTE-158 study. J Clin Oncol.

[22] Heinrich K, Miller-Phillips L, Ziemann F, Hasselmann K, Rühlmann K, Flach M (2023). Lessons learned: the first consecutive 1000 patients of the CCCMunich(LMU) molecular Tumor Board. J Cancer Res Clin Oncol.

[23] Schwartz LH, Seymour L, Litière S, Ford R, Gwyther S, Mandrekar S (2016). RECIST 1.1–Standardisation and disease-specific adaptations: perspectives from the RECIST Working Group. Eur J Cancer.

[24] Pannu HK, Bristow RE, Montz FJ, Fishman EK (2003). Multidetector CT of peritoneal carcinomatosis from ovarian cancer. Radiographics.

[25] D’Angelica M, Gonen M, Brennan MF, Turnbull AD, Bains M, Karpeh MS (2004). Patterns of initial recurrence in completely resected gastric adenocarcinoma. Ann Surg.

[26] Kok P-S, Yoon W-H, Lord S, Marschner I, Friedlander M, Lee CK (2021). Tumor response end points as surrogates for overall survival in immune checkpoint inhibitor trials: a systematic review and meta-analysis. JCO Precision Oncol.

[27] Feinberg BA, Bharmal M, Klink AJ, Nabhan C, Phatak H (2018). Using response evaluation criteria in solid tumors in real-world evidence cancer research. Future Oncol.

[28] Charara RN, Kreidieh FY, Farhat RA, Al-Feghali KA, Khoury KE, Haydar A (2017). Practice and impact of multidisciplinary tumor boards on patient management: a prospective study. J Global Oncol.

[29] Fleissig A, Jenkins V, Catt S, Fallowfield L. Multidisciplinary teams in cancer care: are they effective in the UK? The lancet oncology. 2006;7(11):935–43. 10.1016/S1470-2045(06)70940-8 17081919

[30] Ruhstaller T, Roe H, Thürlimann B, Nicoll JJ (2006). The multidisciplinary meeting: an indispensable aid to communication between different specialities. Eur J Cancer.

[31] Beets G, Sebag-Montefiore D, Andritsch E, Arnold D, Beishon M, Crul M (2017). ECCO essential requirements for quality cancer care: colorectal cancer. A critical review. Crit Rev Oncol/Hematol.

[32] Fassnacht M, Tsagarakis S, Terzolo M, Tabarin A, Sahdev A, Newell-Price J (2023). European Society of Endocrinology clinical practice guidelines on the management of adrenal incidentalomas, in collaboration with the European Network for the study of adrenal tumors. Eur J Endocrinol.

[33] Andritsch E, Beishon M, Bielack S, Bonvalot S, Casali P, Crul M (2017). ECCO essential requirements for quality cancer care: soft tissue sarcoma in adults and bone sarcoma. A critical review. Crit Rev Oncol/Hematol.

[34] Allum W, Lordick F, Alsina M, Andritsch E, Ba-Ssalamah A, Beishon M (2018). ECCO essential requirements for quality cancer care: Oesophageal and gastric cancer. Crit Rev Oncol/Hematol.

[35] Brausi M, Hoskin P, Andritsch E, Banks I, Beishon M, Boyle H (2020). ECCO essential requirements for quality cancer care: prostate cancer. Crit Rev Oncol/Hematol.

[36] Biganzoli L, Cardoso F, Beishon M, Cameron D, Cataliotti L, Coles CE (2020). The requirements of a specialist breast centre. Breast.

[37] Wouters MW, Michielin O, Bastiaannet E, Beishon M, Catalano O, Del Marmol V (2018). ECCO essential requirements for quality cancer care: melanoma. Crit Rev Oncol/Hematol.

[38] Dapper H, Dantes M, Herschbach P, Algül H, Heinemann V. Relevance of tumor boards for the inclusion of patients in oncological clinical trials. J Cancer Res Clin Oncol. 2023:1–8. 10.1007/s00432-022-04559-0 PMC10374816 36995407

