# Correction: Patient eligibility for trials with imaging response assessment at the time of molecular tumor board presentation

**DOI:** 10.1186/s40644-024-00724-5

**Published:** 2024-07-01

**Authors:** Nabeel Mansour, Kathrin Heinrich, Danmei Zhang, Michael Winkelmann, Maria Ingenerf, Lukas Gold, Konstantin Klambauer, Martina Rudelius, Frederick Klauschen, Michael von Bergwelt-Baildon, Jens Ricke, Volker Heinemann, C. Benedikt Westphalen, Wolfgang G. Kunz

**Affiliations:** 1 Department of Radiology, University Hospital, LMU Munich, Marchioninistr. 15, 81377 Munich, Germany; 2 Department of Medicine III, University Hospital, LMU Munich, Munich, Germany; 3 Comprehensive Cancer Center München-LMU (CCCM LMU), LMU Munich, Munich, Germany; 4 Institute of Pathology, Ludwig-Maximilians-Universität Munich, Munich, Germany; 5 German Cancer Consortium (DKTK partner site Munich), Heidelberg, Germany

**Correction: Mansour Cancer Imaging**

10.1186/s40644-024-00708-5

Following publication of the original article [1], we were notified that due to a proofing system error, the author corrections were not submitted. These included mostly typos in the text, as well as the below corrections:

-On page 3, last sentence under Definition of measurable disease, the phrase “Two exemplary cases of MD, which is representative of the overall tumor burden are shown in Fig. 1.” should read “The distribution of MD based on the underlying solid tumor entity is shown in Fig. 1.”

-The caption for Fig. 1 should read “Distribution of measurable based on the underlying tumor entity with highest rate of MD from left to right.” instead of “Distribution of measurable **and non-measurable disease** based on the underlying tumor entity with highest rate of MD from left to right.”

The original article has been corrected.
